# Relationship between estimating glomerular filtration rate and cerebral large artery stenosis: a secondary analysis of a cross-sectional study

**DOI:** 10.3389/fmed.2026.1732178

**Published:** 2026-01-23

**Authors:** Ran Song, Yan Kong, Su-Juan Liu, Wen-Chao Li, Qiang Li, Ming-Huan Yan

**Affiliations:** 1 Department of Radiology, The First Affiliated Hospital of Xinxiang Medical University, Weihui/Xinxiang, China; 2 Department of Ultrasound Medicine, The First Affiliated Hospital of Xinxiang Medical University, Weihui/Xinxiang, China

**Keywords:** cardiovascular risk factors, cross-sectional study, estimated glomerular filtration rate, extracranial arterial stenosis, intracranial arterial stenosis, Korean adults, large cerebral artery stenosis, secondary analysis

## Abstract

**Background:**

Large cerebral artery stenosis (LCAS) is a risk factor for ischemic stroke; however, its relationship with kidney function remains unclear. This study aims to explore the association between glomerular filtration rate (eGFR) and LCAS.

**Methods:**

This is a secondary analysis of a cross-sectional study, with data from 1,011 asymptomatic South Korean adults aged ≥45 years with cardiovascular disease risk factors or stroke family history, recruited from the Neurology Clinic or Health Management Center at CHA Bundang Medical Center in Seoul, between March 2008 and December 2014. The independent variable was eGFR, calculated using the CKD-EPI 2009 creatinine-based equation, and the dependent variable was large cerebral artery stenosis (LCAS), defined as ≥50% stenosis or occlusion of intracranial or extracranial cerebral arteries (or both), assessed by MRA. Multivariable logistic regression was used to evaluate the association between eGFR and LCAS, adjusting for demographic factors, cardiovascular risk factors, and biochemical markers. A generalized additive model (GAM) was used to assess the dose-response relationship between eGFR and LCAS. Stratified analysis and interaction tests were conducted to evaluate the eGFR-LCAS relationship.

**Results:**

Higher eGFR levels were independently associated with lower odds of LCAS. After adjusting for confounding factors, each 1 mL/min/1.73 m^2^ increase in eGFR was associated with a 1% reduction in LCAS risk (OR = 0.99, 95% CI: 0.98–1.00). Compared to the lowest tertile (T1), the highest tertile (T3) was associated with a 39% lower risk of LCAS (OR = 0.61, 95% CI: 0.39–0.96). In subgroup analyses, the association was statistically significant in women (OR = 0.98, 95% CI: 0.96–0.99; *P* = 0.009) and in statin users (OR = 0.97, 95% CI: 0.95–0.99; *P* = 0.015). However, the interaction was borderline for sex (*P* interaction = 0.051) and not significant for statin use (*P* interaction = 0.105), and these subgroup findings should be interpreted as exploratory.

**Conclusion:**

These findings indicate an association between eGFR and LCAS in this cohort. Subgroup patterns and clinical implications require confirmation in prospective and external cohorts. Kidney function may serve as a risk marker; whether it should inform screening strategies requires confirmation. These findings are derived from a South Korean population with cardiovascular risk factors and require validation in diverse populations and general population settings.

## Introduction

Large artery stenosis (LCAS) is a major risk factor for ischemic stroke worldwide. Recent epidemiological studies indicate that the prevalence of symptomatic LCAS is around 10% in Caucasians, while the prevalence of asymptomatic intracranial atherosclerosis can reach up to 50% (1). In Asian populations, the incidence of LCAS is notably higher than in Western populations (2), with LCAS accounting for 33%-67% of strokes or transient ischemic attacks (3). Furthermore, this incidence has been steadily increasing year by year. This trend not only raises stroke risks but also presents a significant challenge to public health systems in Asia.

Estimated glomerular filtration rate (eGFR), a key indicator of kidney function, plays a crucial role in predicting the risk of cardiovascular and cerebrovascular diseases (4, 5). A recent study published in JAMA demonstrates a significant association between reduced eGFR levels and the risk of cardiovascular adverse events, independent of traditional risk factors such as proteinuria (6). This association is evident across different racial populations, though the degree of correlation may vary (7). Research has shown that a decline in eGFR can affect vascular function through various mechanisms, including disturbances in calcium-phosphate metabolism, chronic inflammation, and endothelial dysfunction (8). Notably, these mechanisms appear consistently across different racial groups, suggesting the potential for universally applicable therapeutic targets. A further 2024 study indicates that different eGFR calculation equations may vary in their ability to predict cardiovascular events and all-cause mortality (9). This finding highlights the importance of further exploring the relationship between eGFR and vascular lesions across different populations. However, current research on the relationship between eGFR and LCAS is still limited. Existing studies primarily focus on the correlation between eGFR and intracranial artery stenosis (ICAS) (10–12) and lack sufficient evaluation of extracranial large vessels. Particularly in Asian populations, where the incidence of LCAS is higher, there is a lack of large-scale studies examining the association between eGFR and LCAS. Furthermore, the dose-response relationship between eGFR and LCAS, as well as its variations across different populations, has not been adequately explored.

Based on the current research landscape, we have conducted this retrospective cross-sectional study to explore the relationship between eGFR levels and LCAS in the Korean adult population. This study aims to fill the gap in research on Asian populations and will employ rigorous statistical methods to control for potential confounding factors, thereby enhancing the reliability of the findings. These findings will provide new insights into the relationship between kidney function and cerebrovascular diseases, offering important guidance for the development of clinical preventive strategies.

## Materials and methods

### Study population

This study is a secondary analysis based on the database established by Han-Bin Lee et al., published on November 18, 2015, in *PLoS ONE* (13). The original data were collected from South Korean individuals who visited the Neurology Clinic or the Health Management Center at CHA Bundang Medical Center in Seoul, South Korea, between March 2008 and December 2014. These participants represent a higher-risk population with cardiovascular disease risk factors or stroke family history, rather than the general South Korean population. The inclusion criteria for the study were: (1) age ≥ 45 years; (2) no history of stroke but with cardiovascular disease risk factors or a family history of stroke; (3) having undergone brain MRI and MRA scans. Exclusion criteria included: (1) incomplete medical information; (2) absence of laboratory test results; (3) unclear brain MRI or MRA data affecting evaluation; (4) a history of neurological diseases; (5) occurrence of neurological abnormalities during the study; (6) a history of liver diseases (including active hepatitis, cirrhosis, and liver cancer). All study data were sourced from the hospital's electronic medical records system, including medical records, laboratory test results, and medical imaging data, and were strictly quality-controlled by the original research team. Based on the original database of 1,011 study participants, further statistical analyses were conducted in this study.

This is a secondary analysis of publicly available data from a published research database. This study was carried out in accordance with relevant guidelines and regulations, including the Declaration of Helsinki. The original data collection was ethically approved by the Institutional Review Board of CHA Bundang Medical Center (approval number: BD-2010 083) (13), and written informed consent was obtained from all participants in the original study. For the current secondary analysis, the Ethics Committee of the First Affiliated Hospital of Xinxiang Medical University determined that additional ethical approval was not required for this secondary analysis of publicly available, de-identified data, in accordance with institutional policies governing research using publicly available data.

### Variables

Based on the original study data, this research uses eGFR levels as the primary exposure variable. eGFR was calculated using the Chronic Kidney Disease Epidemiology Collaboration (CKD-EPI) 2009 creatinine-based equation (14), based on serum creatinine measured from fasting venous blood samples collected upon admission. The equation uses sex-specific coefficients without a race coefficient, as applied to an exclusively South Korean population. Cystatin C was not measured in the original study; therefore, cystatin C-based or combined equations were not applicable. Serum creatinine was measured using an enzymatic method on a Hitachi 7600 automatic analyzer (Hitachi High-Technologies Corporation, Tokyo, Japan), with calibration traceable to the isotope dilution mass spectrometry (IDMS) reference standard. Alkaline phosphatase (ALP) was measured using a Hitachi 7600 automatic analyzer. Other laboratory tests, including fasting blood glucose, white blood cells (WBCs), platelets, uric acid, aspartate aminotransferase (AST), alanine aminotransferase (ALT), total cholesterol, and triglycerides, were all performed by the hospital's laboratory using standard methods.

The primary outcome variable of this study is LCAS. All participants underwent 1.5 T brain MRI and MRI angiography. Diagnosis was performed by two experienced radiologists, with further verification by a neurologist. LCAS is defined as a composite outcome of ≥50% stenosis or complete occlusion in ICAS, ECAS, or both. Participants were classified as having LCAS if they had ICAS alone, ECAS alone, or both. ICAS was measured using the WASID method (15), and ECAS was measured using the NASCET method for carotid arteries (16). All MRA images were independently reviewed by two experienced neuroradiologists blinded to clinical data, with discordant readings resolved by consensus. The severity of MS-related cerebral white matter hyperintensities (MS-cWMH) was assessed using the Fazekas scale in conjunction with MRI images.

Hypertension was defined as having a blood pressure ≥140/90 mmHg on repeated measurements or being under antihypertensive treatment. Diabetes was defined as fasting blood glucose ≥126 mg/dL or the use of oral hypoglycemic agents or insulin. Hyperlipidemia was defined as a total cholesterol level ≥240 mg/dL or the use of lipid-lowering medications.

### Statistical analysis

All statistical analyses were conducted using R software version 4.2.0 (R Foundation) and EmpowerStats software (http://www.empowerstats.com, X&Y Solutions, Boston, Massachusetts). Continuous variables are expressed as means ± standard deviation (Mean ± SD), and categorical variables are presented as frequencies and percentages [*n* (%)]. eGFR levels were primarily divided into tertiles for the main analysis. In addition, to enhance clinical interpretability, we conducted a sensitivity analysis using clinically relevant eGFR categories (≥90, 60– < 90, 30– < 60, and < 30 mL/min/1.73 m^2^), with ≥90 mL/min/1.73 m^2^ as the reference group. Categorical variables are presented as *n* (%) and compared using Pearson's chi-square test. For continuous variables, skewed variables are presented as median (interquartile range) and compared using the Kruskal–Wallis test, whereas approximately normally distributed variables are presented as mean ± standard deviation and compared using one-way ANOVA. All statistical tests were two-tailed, with *P* < 0.05 considered statistically significant.

Univariate analysis was performed to assess the impact of various variables on LCAS. Multivariate analysis was then performed to examine the relationship between eGFR levels and LCAS, adjusting for covariates including sex, age, hypertension, diabetes, hyperlipidemia, coronary artery occlusive disease (CAOD), smoking, statin use, ALP, total cholesterol, triglycerides, fasting glucose, and uric acid levels. Covariates were selected based on previous literature, univariate analysis results, and clinical experience, and multicollinearity was assessed using variance inflation factors (VIF); detailed VIF results are provided in Supplementary Table S1. Based on this, three models were developed: the raw model (without any adjustments), Model I (adjusted for demographic factors such as sex and age), and Model II (adjusted for all confounding factors). Subsequently, based on the results of Model II, a generalized additive model was used for smooth curve fitting to analyze the dose-response relationship between eGFR levels and LCAS. Additionally, stratified analysis and interaction tests were performed to evaluate the stability of the relationship between eGFR levels and LCAS.

## Results

This study included 1,011 participants, who were divided into tertiles based on their eGFR levels. The mean eGFR values in the T1, T2, and T3 groups were 56.68 ± 9.92, 73.72 ± 3.53, and 91.86 ± 10.92 mL/min/1.73 m^2^, respectively (*P* < 0.001). The average age showed a decreasing trend from T1 to T3 (68.25 ± 8.21, 63.51 ± 8.65, and 60.74 ± 8.90 years, respectively; *P* < 0.001). Most participants in each group were female, with the highest proportion of females in the T1 group (73.51%). Significant differences were observed between the groups in systolic blood pressure (*P* = 0.022), hematocrit (*P* < 0.001), and uric acid levels (*P* < 0.001). Among comorbidities, hypertension was most prevalent in the T1 group (69.94%), compared to 49.70% in T2 and 52.23% in T3 (*P* < 0.001). Similarly, the prevalence of diabetes was higher in the T1 group (27.08%) than in the T2 group (18.64%) and the T3 group (20.77%; *P* = 0.023). The incidence of MS-cWMH showed a significant decreasing trend across the three groups (T1: 42.26%, T2: 26.92%, T3: 18.10%; *P* < 0.001). The prevalence of cerebral artery stenosis, including ECAS (16.07%, 9.76%, 9.20%; *P* = 0.009), ICAS (13.39%, 9.76%, 6.82%; *P* = 0.017), and LCAS (25.60%, 17.16%, 14.54%; *P* < 0.001), was higher in the T1 group compared to the other groups. The results are shown in Table 1.

**Table 1:** Baseline characteristics of study population according to eGFR tertiles.

**Variables**	**Overall (14.16–140.00)**	**T1 (14.16–67.06)**	**T2 (67.21–78.86)**	**T3 (78.98–140.00)**	***P*-value**
*N*	1,011	336	338	337	
eGFR (mL/min/1.73 m^2^)	74.10 ± 16.81	56.68 ± 9.92	73.72 ± 3.53	91.86 ± 10.92	< 0.001
Age, years	64.16 ± 9.13	68.25 ± 8.21	63.51 ± 8.65	60.74 ± 8.90	< 0.001
**Sex**, ***n*** **(%)**					< 0.001
Male	359 (35.51)	89 (26.49)	121 (35.80)	149 (44.21)	
Female	652 (64.49)	247 (73.51)	217 (64.20)	188 (55.79)	
Smoking, *n* (%)	205 (20.28)	53 (15.77)	76 (22.49)	76 (22.55)	0.043
SBP (mmHg)	131.70 ± 18.32	133.90 ± 18.29	130.17 ± 16.58	131.03 ± 19.80	0.022
DBP (mmHg)	80.04 ± 11.52	79.71 ± 10.49	80.31 ± 11.22	80.09 ± 12.76	0.786
WBC (×109/L)	6.57 ± 1.92	6.75 ± 1.93	6.49 ± 1.96	6.46 ± 1.85	0.101
Hematocrit (%)	40.01 ± 4.13	39.17 ± 4.23	40.50 ± 3.82	40.35 ± 4.20	< 0.001
Platelet (×10^9^/L)	233.15 ± 58.72	236.35 ± 57.29	234.17 ± 60.43	228.93 ± 58.31	0.242
Fasting glucose (mg/dL)	128.04 ± 48.36	128.07 ± 46.27	127.38 ± 49.03	128.66 ± 49.83	0.943
Uric acid (mg/dL)	4.52 ± 1.39	4.93 ± 1.47	4.42 ± 1.28	4.22 ± 1.33	< 0.001
AST (IU/L)	21.00 (18.00–26.00)	22.00 (18.00–27.00)	21.00 (18.00–25.00)	22.00 (18.00–26.00)	0.367
ALT (IU/L)	20.00 (15.00–27.00)	19.00 (15.00–25.00)	20.00 (15.25–27.00)	21.00 (16.00–28.00)	0.131
ALP (IU/L)	183.06 ± 57.98	184.88 ± 59.81	184.70 ± 58.53	179.58 ± 55.53	0.403
Total cholesterol (mg/dL)	193.85 ± 39.82	193.46 ± 42.80	196.05 ± 38.92	192.01 ± 37.58	0.411
Triglyceride (mg/dL)	127.00 (88.00–180.50)	131.50 (91.00–184.00)	129.00 (91.25–189.25)	120.00 (85.00–170.00)	0.044
Hypertension, *n* (%)	579 (57.27)	235 (69.94)	168 (49.70)	176 (52.23)	< 0.001
Diabetes mellitus, *n* (%)	224 (22.16)	91 (27.08)	63 (18.64)	70 (20.77)	0.023
Hyperlipidemia, *n* (%)	332 (32.84)	117 (34.82)	108 (31.95)	107 (31.75)	0.638
CAOD, *n* (%)	52 (5.14)	23 (6.85)	12 (3.55)	17 (5.04)	0.153
Statin medication, *n* (%)	227 (22.45)	77 (22.92)	69 (20.41)	81 (24.04)	0.513
MS-cWMH, *n* (%)	294 (29.08)	142 (42.26)	91 (26.92)	61 (18.10)	< 0.001
SLI, *n* (%)	120 (11.87)	50 (14.88%)	39 (11.54%)	31 (9.20)	0.073
ECAS, *n* (%)	118 (11.67)	54 (16.07)	33 (9.76)	31 (9.20)	0.009
ICAS, *n* (%)	101 (9.99)	45 (13.39)	33 (9.76)	23 (6.82)	0.017
LCAS, *n* (%)	193 (19.09)	86 (25.60)	58 (17.16)	49 (14.54)	< 0.001

eGFR, estimated glomerular filtration rate; SBP, systolic blood pressure; DBP, diastolic blood pressure; WBC, white blood cell; AST, aspartate aminotransferase; ALT, alanine aminotransferase; ALP, alkaline phosphatase; CAOD, coronary artery occlusive disease; MS-cWMH, moderate to severe cerebral white matter hyperintensities; SLI, silent lacunar infarction; ECAS, extracranial arterial stenosis; ICAS, intracranial arterial stenosis; LCAS, large cerebral arterial stenosis. N, number. LCAS is a composite outcome (ICAS and/or ECAS). Among 193 participants with LCAS: 45 had both ICAS and ECAS, 56 had ICAS only, and 92 had ECAS only.

### Univariate analysis

The results of the univariate analysis are presented in Table 2. This study found several factors significantly associated with LCAS. Among these factors, the risk of LCAS was 31% lower in women compared to men (OR = 0.69, 95% CI: 0.50–0.95, *P* = 0.025). For each additional year of age, the risk of LCAS increased by 4% (OR = 1.04, 95% CI: 1.02–1.06, *P* < 0.001). Among cardiovascular disease-related factors, diabetes showed the strongest association, with a 2.45-fold increased risk of LCAS (95% CI: 1.74–3.45, *P* < 0.001). CAOD and hypertension were also significant risk factors, increasing the risk by 137% (OR = 2.37, 95% CI: 1.31–4.30, *P* = 0.004) and 75% (OR = 1.75, 95% CI: 1.25–2.43, *P* = 0.001), respectively. Additionally, for every 1 mg/dL increase in uric acid levels, the risk of LCAS increased by 17% (OR = 1.17, 95% CI: 1.04–1.30, *P* = 0.006). For every 1 mL/min/1.73 m^2^ increase in eGFR, the risk of LCAS decreased by 2% (OR = 0.98, 95% CI: 0.97–0.99, *P* < 0.0001), suggesting that higher kidney function is inversely associated with LCAS risk. Each 1 mg/dL increase in fasting glucose was associated with a 1% increase in the risk of LCAS (OR = 1.01, 95% CI: 1.00–1.01, *P* < 0.001). Notably, eGFR levels showed a clear inverse association with LCAS. Compared to the T1 group, the LCAS risk was reduced by 40% (OR = 0.60, 95% CI: 0.41–0.88, *P* = 0.008) in the T2 group and by 51% (OR = 0.49, 95% CI: 0.34–0.73, *P* < 0.001) in the T3 group, demonstrating a dose-dependent inverse association.

**Table 2:** Univariate analysis of risk factors associated with LCAS.

**Characteristics**	**Total (*n* = 1,011)**	**OR (95% CI)**	***P*-value**
**Sex**, ***n*** **(%)**			0.0248
Male	359 (35.51)	Reference	
Female	652 (64.49)	0.69 (0.50, 0.95)	
Age, years	64.16 ± 9.13	1.04 (1.02, 1.06)	< 0.001
Smoking, *n* (%)	205 (20.28)	0.92 (0.62, 1.36)	0.671
SBP, mmHg	131.70 ± 18.32	1.02 (1.01, 1.02)	< 0.001
DBP, mmHg	80.04 ± 11.52	1.01 (0.99, 1.02)	0.414
Hypertension, *n* (%)	579 (57.27)	1.75 (1.25, 2.43)	0.001
Diabetes mellitus, *n* (%)	224 (22.16)	2.45 (1.74, 3.45)	< 0.001
Hyperlipidemia, *n* (%)	332 (32.84)	1.35 (0.97, 1.87)	0.071
CAOD, *n* (%)	52 (5.14)	2.37 (1.31, 4.30)	0.004
Statin use, *n* (%)	227 (22.45)	1.36 (0.95, 1.94)	0.097
Fasting glucose, mg/dL	128.04 ± 48.36	1.01 (1.00, 1.01)	< 0.001
Uric acid, mg/dL	4.52 ± 1.39	1.17 (1.04, 1.30)	0.006
ALP, IU/L	183.06 ± 57.98	1.00 (1.00, 1.00)	0.595
Total cholesterol, mg/dL	193.85 ± 39.82	1.00 (1.00, 1.00)	0.909
Triglyceride, mg/dL	127.00 (88.00–180.50)	1.00 (1.00, 1.00)	< 0.001
eGFR, mL/min/1.73 m^2^	74.10 ± 16.81	0.98 (0.97, 0.99)	< 0.001
**eGFR, mL/min/1.73 m**^2^ **tertile**			
T1	336 (33.23)	Reference	
T2	338 (33.43)	0.60 (0.41, 0.88)	0.008
T3	337 (33.33)	0.49 (0.34, 0.73)	< 0.001

OR, odds ratio; CI, confidence interval; SBP, systolic blood pressure; DBP, diastolic blood pressure; CAOD, coronary artery occlusive disease; ALP, alkaline phosphatase; eGFR, estimated glomerular filtration rate.

### Multivariable associations between eGFR and LCAS

Multivariable logistic regression analyses were performed using three progressively adjusted models (Table 3). In the non-adjusted model, Lower eGFR was associated with higher odds of LCAS. The direction of association remained consistent in a sensitivity analysis using clinically defined eGFR categories with ≥90 mL/min/1.73 m^2^ as the reference (Supplementary Table S2). This association remained significant after adjusting for age and sex in Model I (OR = 0.98, 95% CI: 0.97–0.99, *P* = 0.005). In the fully adjusted Model II, which controlled for all potential confounders including cardiovascular risk factors, medications, and biochemical markers, the association remained statistically significant (OR = 0.99, 95% CI: 0.98–1.00, *P* = 0.023).

**Table 3:** Multivariate analysis of the associations between eGFR levels and LCAS.

**Variables**	**Non-adjusted model**	***P***	**Model I**	***P***	**Model II**	***P***
eGFR (per mL/min/1.73 m^2^)	0.98 (0.97, 0.99)^***^	< 0.001	0.98 (0.97, 0.99)^**^	0.005	0.99 (0.98, 1.00)^*^	0.023
**eGFR tertile**						
T1	Reference		Reference		Reference	
T2	0.60 (0.41, 0.88)^*^	0.016	0.65 (0.44, 0.96)^*^	0.031	0.72 (0.48, 1.09)	0.052
T3	0.49 (0.34, 0.73)^**^	0.008	0.56 (0.37, 0.84)^*^	0.025	0.61 (0.39, 0.96)^*^	0.044
*P* for trend	0.70 (0.57, 0.85)^**^	0.006	0.74 (0.60, 0.91)^*^	0.020	0.78 (0.62, 0.97)^*^	0.039

LCAS, large cerebral arterial stenosis; eGFR, estimated glomerular filtration rate; OR, odds ratio; CI, confidence interval; CAOD, coronary artery occlusive disease; ALP, alkaline phosphatase.

Data are presented as OR (95% CI).

Model I adjusted for demographic factors (age and sex).

Model II adjusted for factors in Model I plus hypertension, diabetes mellitus, hyperlipidemia, CAOD, smoking status, statin medication, ALP, total cholesterol, triglyceride, fasting glucose, and uric acid levels. BMI (or height/weight) was not available in the shared database and therefore could not be included. ^*^P < 0.05; ^**^P < 0.01; ^***^P < 0.001.

In the tertile analysis, the fully adjusted Model II showed that compared to the lowest tertile (T1), participants in the highest tertile (T3) had a 39% lower odds of LCAS (OR = 0.61, 95% CI: 0.39–0.96, *P* = 0.044), with a significant *P* for trend (OR = 0.78, 95% CI: 0.62–0.97, *P* = 0.039), confirming a dose-dependent inverse association that persisted after comprehensive adjustment.

### Results of the relationship between the eGFR level and LCAS

**Figure 1:**
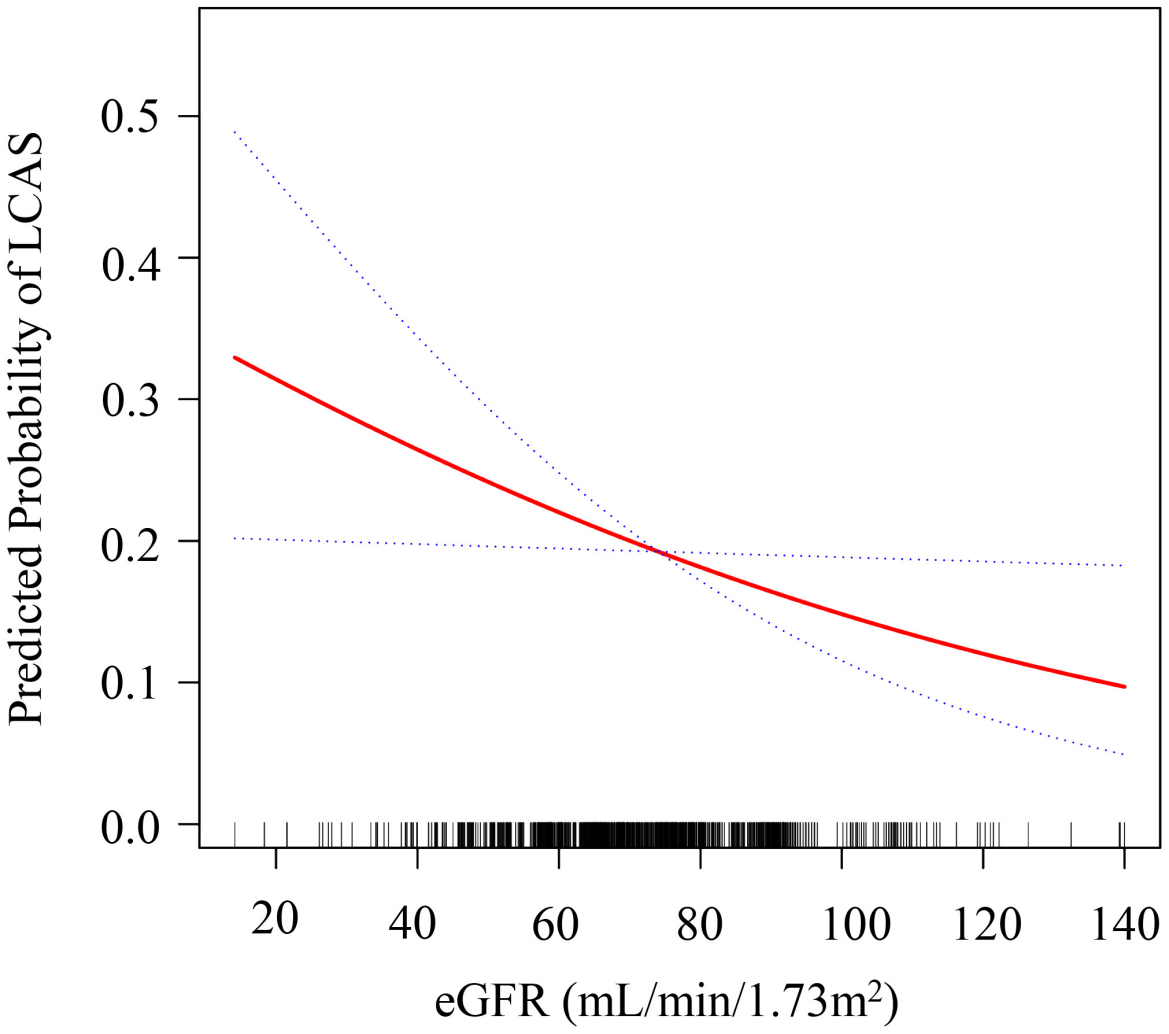
Dose—response plot of estimated glomerular filtration rate (eGFR) vs. predicted probability of large cerebral artery stenosis (LCAS). The x-axis shows eGFR (mL/min/1.73 m^2^), ranging roughly from 15 to 145, and the y-axis shows predicted probability of LCAS from 0.0 to about 0.5. A solid red smooth curve decreases from left to right, indicating lower predicted LCAS probability at higher eGFR. Two blue dotted curves above and below the red line depict the 95% confidence interval. Black rug marks along the x-axis display the distribution of eGFR values.

To assess whether the relationship between eGFR and LCAS is linear or non-linear, we employed a generalized additive model (GAM) with penalized spline smoothing (Figure 1). The smooth term had an estimated degrees of freedom (edf) of 1.0004, approximating 1, which indicates a linear relationship. The likelihood ratio test comparing the GAM to a linear model was not statistically significant (*P* for non-linearity = 0.244), confirming no evidence of non-linear associations. The GAM-fitted curve showed a consistent inverse linear association between eGFR and LCAS across the observed range, supporting a linear dose-response relationship.

### Subgroup analysis

To explore potential effect modification, we performed subgroup analyses after adjusting for potential confounders (Table 4). The results showed a consistent significant association between eGFR and LCAS across most subgroups, with no significant effect modification observed for age (interaction *P* = 0.829), hypertension (interaction *P* = 0.304), diabetes (interaction *P* = 0.677), hyperlipidemia (interaction *P* = 0.533), CAOD (interaction *P* = 0.283), smoking status (interaction *P* = 0.196), and uric acid levels (interaction *P* = 0.973). Notably, we observed a borderline significant effect modification by sex (interaction *P* = 0.051), with women showing a stronger inverse association (OR = 0.98, 95% CI: 0.96–0.99), while no significant association was found in men (OR = 1.00, 95% CI: 0.99–1.02). Additionally, although the interaction did not reach statistical significance (interaction *P* = 0.105), the inverse association between eGFR and LCAS was more pronounced in statin users (OR = 0.97, 95% CI: 0.95–0.99) compared to non-users (OR = 0.99, 95% CI: 0.98–1.01). This suggests that the inverse association between eGFR and LCAS remains stable across different patient subgroups. Although the association was statistically significant within women (*P* = 0.009) and within statin users (*P* = 0.015), the interaction was borderline for sex (*P* interaction = 0.051) and not statistically significant for statin use (*P* interaction = 0.105). Therefore, evidence for effect modification is limited, particularly for statin use. The robustness of this association across various clinical conditions further supports the existence of an independent relationship between kidney function and cerebral arterial stenosis.

**Table 4:** The relationship between eGFR level and LCAS according to different subgroups.

**Subgroup**	**No. of participants**	**OR (95%CI)**	**Interaction *P*-value**
**Sex**			0.051
Male	359	1.00 (0.99, 1.02)	
Female	652	0.98 (0.96, 0.99)	
**Age, years**			0.829
< 60	344	0.99 (0.97, 1.01)	
≥60	667	0.99 (0.97, 1.00)	
**Hypertension**, ***n*** **(%)**			0.304
No	432	0.98 (0.96, 1.00)	
Yes	579	0.99 (0.98, 1.01)	
**Diabetes mellitus**, ***n*** **(%)**			0.677
No	787	0.99 (0.97, 1.00)	
Yes	224	0.99 (0.97, 1.01)	
**Hyperlipidemia**, ***n*** **(%)**			0.533
No	679	0.99 (0.98, 1.00)	
Yes	332	0.98 (0.97, 1.00)	
**CAOD**, ***n*** **(%)**			0.283
No	959	0.99 (0.98, 1.00)	
Yes	52	0.96 (0.91, 1.02)	
**Smoking**, ***n*** **(%)**			0.196
No	806	0.98 (0.97, 1.00)	
Yes	205	1.00 (0.98, 1.03)	
**Statin use**, ***n*** **(%)**			0.105
No	784	0.99 (0.98, 1.01)	
Yes	225	0.97 (0.95, 0.99)	
**Uric acid tertile (mg/dL)**			0.973
Low	302	0.98 (0.96, 1.01)	
Middle	362	0.99 (0.97, 1.01)	
High	339	0.99 (0.97, 1.00)	

## Discussion

In this retrospective cross-sectional study involving 1,011 South Korean participants aged 45 years and older without obvious cranial symptoms, we investigated the association between eGFR and LCAS. The study is characterized by comprehensive clinical data collection and rigorous statistical analysis, utilizing multiple adjusted models. We found that higher eGFR levels are independently associated with lower odds of LCAS, exhibiting a significant dose-dependent relationship (for every 1 mL/min/1.73 m^2^ increase, OR = 0.99, 95% CI: 0.98–1.00). Notably, after adjusting for multiple confounders, the highest tertile (T3) exhibited a 39% lower risk of LCAS compared to the lowest tertile (T1) (OR = 0.61, 95% CI: 0.39–0.96). This inverse association showed consistent findings across various subgroup analyses, particularly in women (OR = 0.98, 95% CI: 0.96–0.99) and statin users (OR = 0.97, 95% CI: 0.95–0.99), where it was even more pronounced.

In our study, the relationship between eGFR and LCAS showed that higher eGFR levels are independently associated with a lower risk of LCAS, exhibiting a significant dose-dependent relationship. Similar findings have been validated in previous studies (17). In a study of 1,703 patients aged over 70 with chronic kidney disease, a decrease in eGFR was associated with an increase in coronary artery calcification (18), suggesting that impaired kidney function may be a significant factor contributing to increased cardiovascular disease risk. Hao et al. (19) found in a cross-sectional study involving 1,762 community-dwelling older adults that kidney dysfunction was significantly associated with intracranial arterial stenosis, and this association was further confirmed through vessel wall magnetic resonance imaging. Similarly, a study by Li et al. (12) involving 5,209 individuals from a Chinese population also demonstrated a strong association between reduced eGFR and asymptomatic intracranial arterial stenosis. Furthermore, a longitudinal study involving 1,627 individuals aged 50–62 from a general population also highlighted the close association between changes in eGFR and the occurrence of cardiovascular events, underscoring the potential importance of monitoring and managing kidney function for cardiovascular health (20). Notably, our findings differ significantly from some previous studies. Song et al. (10) research showed that the association between declining kidney function and intracranial arterial stenosis was more significant in men, which contrasts with the stronger association observed in women in our study. This gender difference may be related to gender-specific variations in endothelial dysfunction (21). Estrogen in women can significantly improve vascular reactivity by modulating endothelial cell function and vascular tone, as well as protect the vascular endothelium by inhibiting the expression of inflammatory factors and oxidative stress responses (22). Additionally, although subgroup estimates suggested a potentially stronger association among statin users, the interaction was not statistically significant (*P* interaction = 0.105). Thus, this observation should be considered hypothesis-generating and requires confirmation. These differences suggest the need for further consideration of the impact of population characteristics, study design, and treatment factors on the research outcomes.

The clinical implications of this association remain to be established, and future prospective studies are warranted to evaluate potential applications. The dose-dependent association between eGFR and LCAS confirmed in this study suggests that maintaining optimal kidney function may be associated with lower cerebrovascular risk, though causality cannot be inferred from our cross-sectional design. Given the high prevalence of chronic kidney disease and cerebrovascular diseases in the elderly population (23), this association is particularly important. Furthermore, the stronger inverse association observed in female patients and statin users provides valuable insights for personalized prevention strategies. In addition, we recommend regular kidney function monitoring for high-risk LCAS patients, especially those with multiple cardiovascular risk factors (24). The results also support the notion that early interventions to preserve kidney function warrant investigation as potential strategies for preventing cerebrovascular complications. Our findings also suggest that patients with reduced eGFR may require more proactive cerebrovascular screening, particularly women and those not currently using statins. Future research should focus on prospective studies to establish causality and explore whether interventions to improve kidney function can effectively prevent or slow the progression of LCAS.

This study has several notable strengths. First, the study employed rigorous statistical methods, using multiple adjustment models to validate the robustness of the findings. A detailed examination of the dose-response relationship was conducted through tertile-based analysis and generalized additive modeling. The GAM analysis confirmed a linear pattern of association (*P* for non-linearity = 0.244, edf = 1.0004), providing deeper insights into the consistent inverse relationship between eGFR and LCAS. Secondly, the extensive subgroup analyses in this study confirmed the stability of the relationship, offering new insights into this field. However, several limitations of this study need to be addressed. First, this single-center study has important generalizability limitations. Participants were recruited from a Neurology Clinic and Health Management Center with cardiovascular risk factors or stroke family history, representing a higher-risk population rather than the general population. Findings should not be generalized to healthy individuals or the general South Korean population. Second, as the study was conducted exclusively in South Koreans, findings may not apply to other ethnic groups (including Chinese or other Asian populations) due to differences in genetic susceptibility, lifestyle factors, and healthcare systems. Multi-ethnic validation studies are needed. Second, the cross-sectional design of the study only allows for the assessment of the association between eGFR and LCAS, without establishing causality. Third, although we adjusted for several measurable confounders, unmeasured factors such as dietary habits, physical activity levels, and genetic influences may have affected the results. BMI (and height/weight) was not available in the shared database; therefore, we could not adjust for adiposity-related confounding, and residual confounding may persist. Furthermore, multiple subgroup analyses were performed; therefore, subgroup findings should be interpreted cautiously as exploratory and not as confirmatory evidence of effect modification.

## Conclusion

This study identified an independent inverse association between eGFR and large cerebral artery stenosis (a composite of intracranial and extracranial stenosis) in South Korean adults with cardiovascular risk factors or a family history of stroke. A dose–response pattern was observed across eGFR levels. Subgroup analyses suggested potentially stronger associations in women and in statin users; however, these subgroup findings should be interpreted cautiously as exploratory. Given the cross-sectional design, our results indicate statistical associations rather than causal relationships. Kidney function may serve as a potential risk marker in this specific population, but whether eGFR monitoring should inform screening or prevention strategies requires confirmation in prospective and external cohorts. The findings should not be generalized to the general South Korean population or other ethnic groups without further validation. Future studies are warranted to clarify underlying mechanisms and to formally test effect modification by sex and statin use.

## Data Availability

The datasets presented in this study can be found in online repositories. The names of the repository/repositories and accession number(s) can be found below: This study is a secondary analysis based on the database established by Lee et al., published on November 18, 2015, in PLoS ONE (13).

## References

[1] Panagiotopoulos E Stefanou MI Magoufis G Safouris A Kargiotis O Psychogios K . Prevalence, diagnosis and management of intracranial atherosclerosis in White populations: a narrative review. Neurol Res Pract. (2024) 6:54. doi: 10.1186/s42466-024-00341-4 39523357 PMC11552123

[2] Liu M Sariya S Khasiyev F Tosto G Dueker ND Cheung YK . Genetic determinants of intracranial large artery stenosis in the northern Manhattan study. J Neurol Sci. (2022) 436:120218. doi: 10.1016/j.jns.2022.120218 35259553 PMC9018518

[3] Wang Y Zhao X Liu L Soo YO Pu Y Pan Y . Prevalence and outcomes of symptomatic intracranial large artery stenoses and occlusions in China: the Chinese Intracranial Atherosclerosis (CICAS) Study. Stroke. (2014) 45:663–9. doi: 10.1161/STROKEAHA.113.003508 24481975

[4] Abenavoli C Provenzano M Ksiazek SH Hu L Cuna V Manna G . Role of estimated glomerular filtration rate in clinical research: the never-ending matter. Rev Cardiovasc Med. (2024) 25:1. doi: 10.31083/j.rcm2501001 39077647 PMC11262368

[5] Matsushita K Coresh J Sang Y Chalmers J Fox C Guallar E . Estimated glomerular filtration rate and albuminuria for prediction of cardiovascular outcomes: a collaborative meta-analysis of individual participant data. Lancet Diabetes Endocrinol. (2015) 3:514–25. doi: 10.1016/S2213-8587(15)00040-6 26028594 PMC4594193

[6] Grams ME Coresh J Matsushita K Ballew SH Sang Y Surapaneni A . Estimated glomerular filtration rate, albuminuria, and adverse outcomes: an individual-participant data meta-analysis. JAMA. (2023) 330:1266–77. doi: 10.1001/jama.2023.17002 37787795 PMC10548311

[7] Tang H Chen W Bian J O'Neal LJ Lackland DT Schatz DA . Ethnic variations in cardiovascular and renal outcomes from newer glucose-lowering drugs: a meta-analysis of randomized outcome trials. J Am Heart Assoc. (2023) 12:e026791. doi: 10.1161/JAHA.122.026791 37158069 PMC10227303

[8] Inker LA Grams ME Levey AS Coresh J Cirillo M Collins JF . Relationship of estimated GFR and albuminuria to concurrent laboratory abnormalities: an individual participant data meta-analysis in a global consortium. Am J Kidney Dis. (2019) 73:206–17. doi: 10.1053/j.ajkd.2018.08.013 30348535 PMC6348050

[9] Wang H Cai J Fan H Diamantidis CJ Young BA Bidulescu A. Prediction of cardiovascular events and all-cause mortality using race and race-free estimated glomerular filtration rate in African Americans: the Jackson Heart Study. Front Med. (2024) 11:1432965. doi: 10.3389/fmed.2024.1432965 39544376 PMC11560791

[10] Song X Li J Hua Y Wang C Liu B Liu C . Chronic kidney disease is associated with intracranial artery stenosis distribution in the middle-aged and elderly population. J Atheroscler Thromb. (2020) 27:245–54. doi: 10.5551/jat.49569 31462617 PMC7113140

[11] Kang K Hwang YH. The relationship between intracranial arterial stenosis and glomerular filtration rate. J Thromb Thrombolysis. (2012) 34:310–7. doi: 10.1007/s11239-012-0739-1 22618152

[12] Li Z Li J Wang A Pan H Wu S Zhao X. Decreased estimated glomerular filtration rate (eGFR), not proteinuria, is associated with asymptomatic intracranial arterial stenosis in chinese general population. Sci Rep. (2017) 7:4619. doi: 10.1038/s41598-017-04549-0 28676650 PMC5496910

[13] Lee HB Kim J Kim SH Kim S Kim OJ Oh SH. Association between serum alkaline phosphatase level and cerebral small vessel disease. PLoS ONE. (2015) 10:e0143355. doi: 10.1371/journal.pone.0143355 26580067 PMC4651565

[14] Levey AS Stevens LA Schmid CH Zhang YL Castro AF Feldman HI . A new equation to estimate glomerular filtration rate. Ann Intern Med. (2009) 150:604–12. doi: 10.7326/0003-4819-150-9-200905050-00006 19414839 PMC2763564

[15] Samuels OB Joseph GJ Lynn MJ Smith HA Chimowitz MI. A standardized method for measuring intracranial arterial stenosis. AJNR Am J Neuroradiol. (2000) 21:643–6. 10782772 PMC7976653

[16] North American Symptomatic Carotid Endarterectomy Trial Collaborators Barnett HJM Taylor DW Haynes RB Sackett DL Peerless SJ . Beneficial effect of carotid endarterectomy in symptomatic patients with high-grade carotid stenosis. N Engl J Med. (1991) 325:445–53. 1852179 10.1056/NEJM199108153250701

[17] Hyun YY Kim H Oh KH Ahn C Park SK Chae DW . eGFR and coronary artery calcification in chronic kidney disease. Eur J Clin Invest. (2019) 49:e13101. doi: 10.1111/eci.13101 30866052

[18] El Barzouhi A Elias-Smale S Dehghan A Vliegenthart-Proença R Oudkerk M Hofman A . Renal function is related to severity of coronary artery calcification in elderly persons: the Rotterdam study. PLoS ONE. (2011) 6:e16738. doi: 10.1371/journal.pone.0016738 21311747 PMC3032739

[19] Hao Q Gottesman RF Qiao Y Liu L Sharma R Selvin E . Association between kidney disease measures and intracranial atherosclerosis: the ARIC study. Neurology. (2020) 94:e2361–72. doi: 10.1212/WNL.0000000000009311 32303651 PMC7357292

[20] Melsom T Norvik JV Enoksen IT Stefansson V Rismo R Jenssen T . Association of high-density lipoprotein cholesterol with GFR decline in a general nondiabetic population. Kidney Int Rep. (2021) 6:2084–94. doi: 10.1016/j.ekir.2021.05.007 34386657 PMC8343778

[21] Kelly DM Ademi Z Doehner W Lip GYH Mark P Toyoda K . Chronic kidney disease and cerebrovascular disease: consensus and guidance from a KDIGO controversies conference. Stroke. (2021) 52:e328–46. doi: 10.1161/STROKEAHA.120.029680 34078109

[22] Ueda K Fukuma N Adachi Y Numata G Tokiwa H Toyoda M . Sex differences and regulatory actions of estrogen in cardiovascular system. Front Physiol. (2021) 12:738218. doi: 10.3389/fphys.2021.738218 34650448 PMC8505986

[23] Yuan G Yang Y Lin Y Lin J Wu Y. Current status and development trends in CKD with frailty research from 2000 to 2021: a bibliometric analysis. Ren Fail. (2024) 46:2292142. doi: 10.1080/0886022X.2023.2292142 38178378 PMC10773684

[24] Zeng G Zhu P Yuan D Wang P Li T Li Q . Clin Kidney J. (2024) 17:sfae032. doi: 10.1093/ckj/sfae032 38435350 PMC10906361

