# Supplementary material for: Relationship between estimating glomerular filtration rate and cerebral large artery stenosis: a secondary analysis of a cross-sectional study
Source: Front Med (Lausanne). 2026 Jan 23;13:1732178. doi: 10.3389/fmed.2026.1732178 (PMC12875958; doi:10.3389/fmed.2026.1732178)
Supplement: Supplementary file 1 [file Table_1.docx]

Supplementary Table S1. Variance inflation factor (VIF) diagnostics for covariates included in the fully adjusted model

| **Covariate** | **VIF** |
| --- | --- |
| eGFR (ml/min/1.73m²) | 1.3 |
| Sex | 1.6 |
| Age (years) | 1.2 |
| Hypertension | 1.1 |
| Diabetes mellitus | 1.2 |
| Hyperlipidemia | 2.9 |
| CAOD | 1.1 |
| Smoking | 1.3 |
| Statin medication | 2.3 |
| Fasting glucose (mg/dL) | 1.2 |
| Uric acid (mg/dL) | 1.3 |
| ALP (IU/L) | 1.0 |
| Total cholesterol (mg/dL) | 1.5 |
| Triglycerides (mg/dL) | 1.2 |
