# Supplementary material for: Relationship between estimating glomerular filtration rate and cerebral large artery stenosis: a secondary analysis of a cross-sectional study
Source: Front Med (Lausanne). 2026 Jan 23;13:1732178. doi: 10.3389/fmed.2026.1732178 (PMC12875958; doi:10.3389/fmed.2026.1732178)
Supplement: Supplementary file 2 [file Table_2.docx]

Supplementary Table S2. Association between clinically defined eGFR categories and LCAS

| **eGFR category (mL/min/1.73m²)** | **Non-adjusted OR (95% CI), *P*** | **Model I OR (95% CI), *P*** | **Model II OR (95% CI), *P*** |
| --- | --- | --- | --- |
| ≥90 | Reference | Reference | Reference |
| 60–<90 | 1.60 (0.97, 2.67), 0.0675 | 1.36 (0.81, 2.30), 0.2445 | 1.36 (0.79, 2.33), 0.2622 |
| 30–<60 | 2.19 (1.22, 3.94), 0.0086 | 1.63 (0.86, 3.07), 0.1312 | 1.45 (0.75, 2.78), 0.2683 |
| <30 | 6.90 (1.83, 25.97), 0.0043 | 5.87 (1.52, 22.60), 0.0102 | 4.44 (1.08, 18.23), 0.0385 |
| *P* for trend | 1.54 (1.19, 2.00), 0.0010 | 1.38 (1.04, 1.83), 0.0270 | 1.27 (0.95, 1.70), 0.1081 |

*Notes:* Model I adjusted for demographic factors (age and sex).

Model II adjusted for factors in Model I plus hypertension, diabetes mellitus, hyperlipidemia, CAOD, smoking status, statin medication, ALP, total cholesterol, triglyceride, fasting glucose, and uric acid levels (consistent with the tertile models).
